# Surveillance of noise exposure level in industrial enterprises—Jiangsu Province, China, 2022

**DOI:** 10.3389/fpubh.2024.1230481

**Published:** 2024-02-12

**Authors:** Cuicui Zhang, Jianfeng Wang, Huan Wang, Hengdong Zhang

**Affiliations:** Jiangsu Provincial Center for Disease Control and Prevention, Nanjing, China

**Keywords:** noise, exposure level, industrial enterprises, Jiangsu Province, China

## Abstract

Occupational noise exposure is the most prominent problem in industrial enterprises in Jiangsu Province. Since 2019, China has established the National Surveillance System for Occupational Hazards in the workplace to grasp the current occupational hazards in critical industries, including occupational noise. According to the *Work Plan for Surveillance of Occupational Hazards in the Workplace (2022)* issued by the National Health Commission of the People’s Republic of China, the noise exposure level of 3,142 enterprises in our province was monitored, the median and interquartile range (IQR) were calculated, and the distribution of noise exposure level was described by industry classification, enterprise-scale and ownership type of the enterprise. The railway, shipping, aerospace, and other transportation equipment manufacturing industries exhibited the highest proportion (42.9%) of individual noise exposure levels exceeding 85 dB(A), followed by the motor vehicles manufacturing industry (36.4%). The proportion of individual noise exposure levels exceeding 85 dB(A) was higher in medium and small enterprises, with rates of 28.1 and 28.6%, respectively. The highest proportion of personal noise exposure levels exceeding 85 dB(A) was observed in Hong Kong, Macao and Taiwan investment enterprises (37.5%), followed by incorporated companies (34.6%) and limited liability companies (28.1%), the lowest was state-owned enterprises(15.5%). The areas with excessive noise are primarily concentrated in grinding, welding, machining, cutting, and other related operations, accounting for 61.2% of the total. Among these operations, grinding accounts for 29.8%. The highest environmental noise and individual noise intensity were found in sandblasting and grinding positions, with individual noise intensities of 115.5 dB(A) and 108.4 dB(A), respectively. The noise exposure risk is so high that cannot be ignored in the manufacturing industry, especially in Hong Kong, Macao, and Taiwan investment enterprises, incorporated companies and medium and small enterprises.

## 1 Introduction

More than 10% of workers in highly industrialized cultures have occupational noise-induced hearing loss (1–3). Long-term noise exposure not only leads to hearing loss, but also induces detrimental effects on the nervous system, digestive system, cardiovascular system, and other physiological systems in humans (4–6). Recent research findings indicate that noise-induced hypertension, ischemic heart disease, sleep disorders, and prolonged noise exposure can also contribute to the development of learning disabilities and anxiety, as well as discomfort and other adverse psychological conditions (7–11). Occupational diseases in China are different from other diseases, which have clear definitions, classifications and characteristics. Occupational diseases refer to diseases caused by exposure to dust, radioactive substances and other toxic and harmful factors in the occupational activities of workers in enterprises, institutions and individual economic organizations. Occupational diseases have the following five characteristics: (1) the etiology is clear and specific; (2) most of the etiologies can be detected, generally showing a dose–response relationship; (3) the same factor exposed population has a certain incidence, individual cases are few; (4) early diagnosis, reasonable treatment and a better prognosis, but only for individual treatment is not helpful to protect the health of the people still in contact with; and (5) most of the occupational diseases still lack effective treatment and preventive measures, should strengthen the protection of people’s health. Following pneumoconiosis, noise-induced hearing loss has emerged as the second most prevalent occupational ailment in China over the past 4 years (12). More than thirty million workers in China are exposed to noise (13, 14). Jiangsu Province has developed economy and numerous industrial enterprises, so the monitoring of noise exposure level in Jiangsu Province is more representative. Yu Bin’s research revealed that the manufacturing industry accounted for 93.5% of suspected cases of occupational noise-induced hearing loss among the seven major industries in Jiangsu Province. The industries with higher detection rates included the production of coke and refined petroleum products; the manufacturing of furniture; metal products; railway equipment, shipping equipment, aerospace equipment, and other transportation equipment; as well as the production of leather, fur, feathers and their products, and the footwear industry (15). The incidence of noise-induced hearing loss in Jiangsu Province is the highest among occupational diseases in 2022 (16). Therefore, it is necessary to understand the current situation of noise hazards in various industries in Jiangsu Province, to study and analyze the noise distribution and intensity levels in workplaces of varying sizes and types of employers, and to provide the scientific basis for supervision and law enforcement, research and revision of occupational disease prevention and control regulations, standards, and guidelines.

## 2 Object and methods

### 2.1 Object

To estimate the overall noise exposure level and development trend of industrial enterprises in the province, 3,142 enterprises were selected from the database of the National Surveillance System for Occupational Hazards in the workplace in 2022 and classified according to industry classification, enterprise size and ownership type.

### 2.2 Methods

#### 2.2.1 Monitoring of industry and regional distribution

According to the *Work Plan for Surveillance of Occupational Hazards in the Workplace (2022)* issued by National Health Commission of the People’s Republic of China and the current situation in Jiangsu Province, 13 districts and cities should be monitored, and the coverage rate of counties (cities, districts) carrying out monitoring of occupational hazards in workplaces reaches 100%. A minimum of 2,850 enterprises should be monitored, with the average number of businesses in each county not falling below 30. The Work Plan for Surveillance of Occupational Hazards in the Workplace (2022) identifies the key industries and the specific small and medium-sized industries to be monitored, which are based on the monitoring of occupational hazard factors in the workplace for 2019–2021 and the monitoring of key occupational diseases (17).

#### 2.2.2 Monitoring sites and post requirements

The noise exposure levels of environmental and individual workplaces in each enterprise were measured. Environmental noise represents the real-time noise intensity of the workplace, while individual noise represents the actual noise exposure level of the workplace, including all ambient noise during daily activities such as noon work and rest. The purpose of citing environmental noise is to visually see the real-time noise intensity, compare the noise intensity of enterprises in different industries, economic types and sizes, and analyze its relationship with individual noise levels from the side. According to the size of the employer, the measurement quantity requirements of the noise contact posts and working places are as follows: (1) For large and medium-sized enterprises, each employer shall select no less than 4 posts in contact with noise for monitoring, and measure the noise intensity of all working places involved in the monitoring positions (in principle, select working sites above 80 dB(A)); (2) All noisy jobs and work locations should be measured for small and mini-sized enterprises.

#### 2.2.3 Noise measurement method

The noise measurement requires a sound level meter of at least type 2, equipped with A-weighting and slow response settings. The 8 h equivalent A sound level (LEX,8 h) or 40 h equivalent A sound level (LEX, W) of the noise exposure position are measured by the corresponding measurement method according to the operation mode: (1) When the noise exposure of the entire work shift is regular, the 8 h equivalent A sound level (LEX,8 h) of the position can be calculated according to the noise intensity of the fixed work place and the noise exposure time of each shift, or the calculation can be made by individual noise measurement. If the work week is not 5 days, the equivalent A sound level (LEX, W) results need to be translated into 40 h; (2) When workers work in irregular locations or work shifts are exposed to noise irregularly, individual noise measurement shall be adopted, and the duration of individual measurement shall not be less than 50% of the actual working time of each work shift. Ensure that the measurement time has covered all the work content of contact noise, and calculate the 8 h equivalent A sound level or 40 h equivalent A sound level of the position according to the contact time (LEX, 8 h/LEX, W); and (3) the sampling method and noise measure method for both environmental and individual noise exposure were determined in accordance with the *Measurement of Physical Agents in the Workplace Part 8: Noise (GBZ/T189.8–2007).*

#### 2.2.4 Quality control

The Jiangsu Provincial Center for Disease Control and Prevention shall be responsible for formulating the quality control plan for the province’s surveillance work, and the monitoring project undertaking institutions at all levels shall carry out the monitoring work in accordance with the requirements of unified methods, unified standards and unified control; All technical personnel involved in monitoring work shall participate in operational training organized by provincial or municipal monitoring agencies to ensure the unity, integrity and standardization of monitoring data. Jiangsu Provincial Center for Disease Control and Prevention shall select no less than 40 employers for on-site verification, and select 10% of the total number of monitoring employers to carry out laboratory original record review. The selected employers shall cover the monitoring municipal units and all key industry types within their jurisdiction. The municipal quality control agency shall extract 10% of the monitoring employers for on-site verification, and the extracted employers shall cover all county-level units that carry out monitoring work within the jurisdiction.

### 2.3 Statistics

Using SPSS 21.0, the median and interquartile range (IQR) were computed, and the distribution of noise exposure was defined by industry classification, enterprise size, and economical kind of enterprise. To evaluate noise exposure levels across various dimensions, the Kruskal-Wallis H test was utilized. The level of significance for testing was *p* < 0.05.

## 3 Results

### 3.1 Environmental noise and individual noise monitoring results

**Figure 1:**
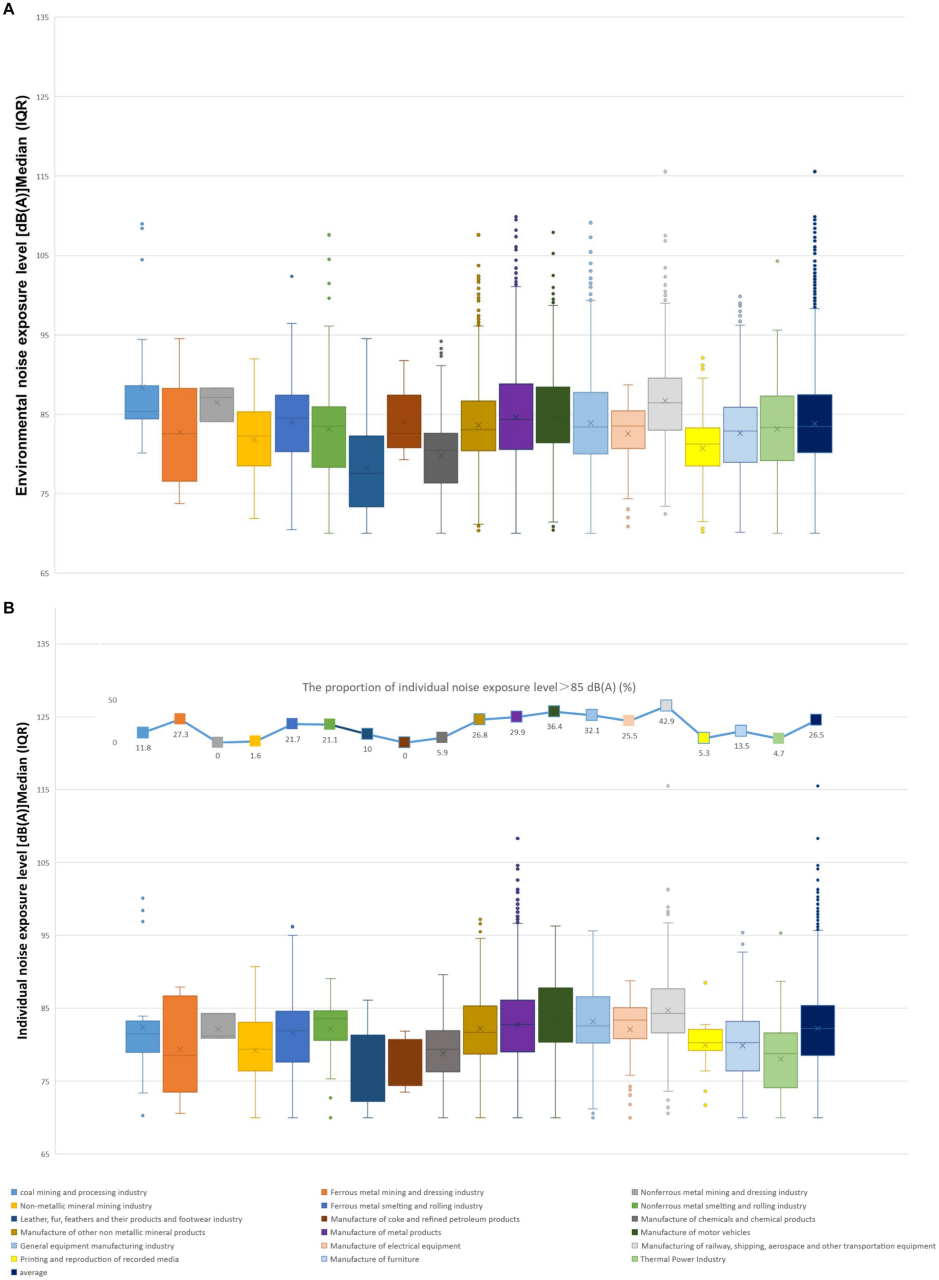
Environmental noise exposure levels **(A)** and individual noise exposure levels **(B)** among the industrial enterprises – Jiangsu Province, China, 2022.

7,746 environmental noise samples and 3,676 individual samples were detected in this survey of 3,142 businesses across essential industries, as shown in Figure 1. The environmental noise samples and individual noise samples were subjected to statistical analysis within each category in order to determine the median and quartile values for each respective category.

Overall, 26.5% of the individual noise exposure samples exceeding the Chinese national standard Railway, shipping, aerospace and other transportation equipment manufacturing industry had the most significant proportion of noise exposure levels exceeding 85 dB(A) at 42.9%, followed by the production of motor vehicles, nonferrous metal smelting and rolling sector, 36.4 and 32.1%, respectively (Figure 1B). The surveillance results revealed that the environmental noise exposure medians were greater than 85 dB(A) in the coal mining and processing, Nonferrous metal mining and dressing, railway, shipping, aerospace, and other transportation equipment manufacturing industries. In comparison, the individual noise exposure medians were less than 85 dB(A). The railway, shipping, aerospace and other transportation equipment manufacturing industry had the highest median individual noise exposure levels at 84.3 dB(A) (Figure 1A).

The median environmental noise exposure level at the chosen workplace was 83.5 dB(A), and the median individual noise exposure level was 82.2 dB(A). The railway, shipping, aerospace and other transportation equipment manufacturing, motor vehicles, and metal products industries have a proportion of individual noise exposure that exceeds the average level of 26.5%. The environmental noise exposure levels in Nonferrous metal mining and dressing industry, railway, shipping, aerospace and other transportation equipment manufacturing industry, coal mining and processing, motor vehicles manufacturing industry, ferrous metal smelting and rolling industry, metal products industry, electrical equipment manufacturing industry, nonferrous metal smelting and rolling industry were higher than the total level, with a median and interquartile range of 87.1 (85.6, 87.7), 86.4 (83.1, 89.5), 85.4 (84.4, 87.9), 84.5 (81.4, 88.4), 84.5 (80.3, 87.4), 84.3 (80.6, 88.8), 83.5 (80.8, 85.2), 83.5 (78.4, 85.9), respectively (Figure 1A). The disparity between environmental and individual noise exposure medians in the coke and refined petroleum products industries was 6.3 dB(A), which was more higher than other divisions.

### 3.2 Noise monitoring results of different enterprise sizes and economic types

**Figure 2:**
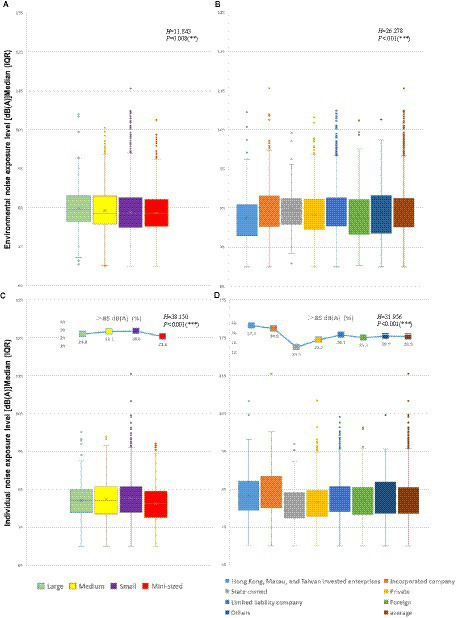
The levels of environmental noise exposure among industrial enterprises vary based on enterprise-scale **(A)** and ownership types **(B)**, as well as the individual noise exposure levels in different enterprise-scale **(C)** and ownership types **(D)** Jiangsu Province, China, 2022.

More medium and small enterprises reported individual noise exposure levels exceeding 85 dB(A) (Figure 2). Large enterprises had the most remarkable environmental noise median (Figures 2A,B), whereas small enterprises had the highest individual noise median (Figures 2C,D). The largest median difference between the environmental and individual noise exposure levels in large businesses was 2.3 dB(A). Figure 2 also depicts the distribution of noise exposure levels among enterprise ownership categories. Individual noise exposure levels over 85 dB(A) were the lowest in State-owned enterprises. The highest individual noise exceeding standard rate was found in Hong Kong, Macao and Taiwan investment enterprises, incorporated companies, limited liability companies and other economic types, which were 37.5, 34.6, 28.1, and 26.9%, respectively (Figures 2C,D). Furthermore, the median values of individual noise for these economic types also exceed the average level of 26.5%.

## 4 Discussion

Surveillance of occupational hazards in the workplace has been carried out for 4 years by the Chinese government since 2019. Through the nationwide surveillance of occupational disease hazard factors in the workplace of employers, the Chinese government has mastered the current situation of occupational disease hazards and assessed the impact of exposure to occupational disease hazard factors and positions on the health of laborers in critical industries. This study mainly analyzes the noise monitoring results of industrial enterprises in Jiangsu Province in 2022. 97.8% of the 3,142 enterprises selected were from the manufacturing industry, and the proportion of noise exposure levels exceeding 85 dB(A) was still high. The median of individual noise exposure levels in all industries exceeded 80 dB(A) except the Leather, fur, feathers and their products and footwear industry, coke and refined petroleum products industry, chemicals and chemical products manufacturing industry and electricity and heat production and supply industry. According to the Classification of Occupational Hazards at Workplaces Part 4: Occupational Exposure to Noise (GBZ/T 229.4-2012) (18), more than 68% of the selected 3,142 enterprises have noise exposure.

The surveillance results showed that the median individual noise exposure level in railway, shipping, aerospace, and other transportation equipment manufacturing industries was the highest, followed by the motor vehicles manufacturing industry, nonferrous metal smelting, and rolling industry. The overall noise exposure level of the manufacturing industry was higher than that of other industries, which was consistent with the conclusion that the industry with the most extensive distribution of new occupational diseases was the manufacturing industry in the critical occupational disease monitoring system of Jiangsu Province in 2022 (16). This is also consistent with the 2014 report on noise exposure in Malaysian industrial enterprises. The industries with excessive noise in Malaysian industrial enterprises are concentrated in manufacturing, and the proportion of workers exposed to occupational noise in metal products industry is the highest (19). Liu Jing’s research on the status quo of noise hazards in an industrial area in Tianjin pointed out that the noise exceeding rate of different industries was quite different, and the high-noise industries were metal products, transportation equipment manufacturing (20). The areas of operation where the noise levels exceeded national standard limits were concentrated in grinding, welding, machining, cutting, and other posts, accounting for 61.2% of the total. Among these operations, grinding accounts for 29.8%. This finding is basically consistent with Zheng Jiao’s analysis of the noise monitoring results obtained from industrial enterprises in Feicheng City, Shandong Province in 2022 (21). The highest environmental noise and individual noise intensity were found in sandblasting and grinding positions, with individual noise intensities of 115.5 dB(A) and 108.4 dB(A), respectively. Some studies have shown that automobile manufacturing, metal processing, shipbuilding, electronics, machinery, and other manufacturing industries, work forging, stamping, welding, grinding, and so on are specific industries and types of work that produce non-steady noise. The probability and severity of occupational noise deafness in these types of work were higher than those of workers exposed to steady noise (22). The above posts exceed the standard primarily due to the collision between grinding machines, welding machines, cutting machines, and metal components. The welding and grinding positions are primarily concentrated and abundant, resulting in a potential for significant noise intensity amplification due to the overlapping effects of multiple independent sound sources. Of course, the reasons for the high level of noise exposure also include the rotation and vibration of noise equipment, unreasonable equipment layout, and lack of effective sound insulation and noise reduction measures. The engineering measures to control mechanical noise are very complicated. It is recommended to set up the equipment sound shield and silencer, rationally arrange the equipment, and strengthen the personal protection. The implementation of personal protective measures is crucial in preventing occupational diseases, particularly by providing appropriate hearing protection to individuals exposed to excessive noise. Currently, this approach is widely adopted in enterprises as the most cost-effective means of safeguarding and has become the prevailing practice. 67% of the selected railway, shipping, aerospace and other transportation equipment enterprises were shipbuilding and motorcycle parts and accessories manufacturing industries, and 81% were small and mini-sized enterprises. In addition, Poor self-protection awareness and poor management and technology investment resulted in severe hearing loss of workers in small and mini-sized enterprises. Another study showed that noise exposure was the most direct factor for high-frequency hearing loss (23). The increased binaural high-frequency hearing threshold rate in motor vehicles, railway, shipping, aerospace and other transportation equipment manufacturing industry was also high in the surveillance system of key occupational diseases in Jiangsu Province in 2022 (16). More studies have shown that occupational noise exposure was more evident with the increase in working years. Another study showed that combined exposure of ototoxic substances and noise was more likely to cause hearing loss than exposure at the same exposure level (24).

The analysis of the relationship between noise exposure levels and different enterprise scales showed that medium and small enterprises had a higher proportion of individual noise exposure levels exceeding 85 dB(A) than large and mini-sized enterprises. Relevant studies showed that the occupational contraindications detection rate for noise-exposed workers was higher in medium and small enterprises than in large and mini-sized enterprises in Jiangsu Province (15). In the critical occupational disease monitoring system of Jiangsu Province in 2022, the incidence of medium and small enterprises was the highest, and that of large enterprises was the lowest. The noise monitoring conducted by Zhou Kai in Puyang City revealed that the predominant presence of noise hazards lies within small-scale enterprises (49.22%), characterized by high levels of noise intensity (25). This phenomenon is mainly because most of the leaders and managers of medium and small enterprises had relatively weak awareness of occupational disease prevention and control, ignored the importance of occupational disease prevention and control, and insufficient investment in occupational disease protection facilities, which had a variety of occupational health problems (15, 25–29). The surveillance results also showed that the proportion of individual noise exposure levels exceeding 85 dB(A) in state-owned enterprises was much lower than in other enterprises. The proportion of individual noise exposure levels exceeding 85 dB(A) and the noise median was higher in Hong Kong, Macao and Taiwan investment enterprises, incorporated enterprises and limited liability companies. This is because the large state-owned enterprises have full legal awareness and can strictly implement the relevant provisions of the occupational disease prevention and control law and higher occupational disease prevention and control investment and management level. The individual noise exposure level of Hong Kong, Macao, and Taiwan investment enterprises, Incorporated enterprises, and limited liability companies was 1.1 ~ 1.8 dB(A) higher than that of state-owned enterprises, which is related to the low attention and insufficient investment of the above enterprises in the prevention and treatment of occupational diseases (30).

## 5 Conclusion

This is a comprehensive report on the noise exposure level of occupational hazards in workplaces in Jiangsu Province based on different industries, enterprise scales, and ownership types in 2022. The noise exposure risk of critical industries, especially manufacturing, was still high, threatening the health of many workers. If enterprises still pay too much attention to economic development and ignore the tendency of workers’ health, which seriously violates the two core directions of disease prevention and health promotion in *Healthy China Action (2019–2030)* (31). In that case, the detailed surveillance of noise exposure levels will provide a basis for the occupational health supervision department to conduct targeted supervision and formulate special control measures for industries and positions with serious hazards and the 14th Five-Year Plan for Occupational Disease Prevention and Control of Jiangsu Province. More attention should be paid to the supervision of small and medium-sized enterprises and non-state-owned enterprises. Special efforts should be made to control noise in key industries such as the automobile manufacturing industry, railway, ship, aerospace, and other transportation equipment manufacturing industries. The noise should be controlled by improving the automation level of process equipment, taking effective measures of sound absorption, sound insulation and vibration reduction, reasonable layout of equipment, working time control, personnel protection and other measures. In addition, it is important to consider the process feasibility and economic rationality. This study’s limitation is that some non-fixed-point operation posts or non-steady-state noise posts were not strictly monitored by individual measurement methods, the quality of monitoring work needs to be further improved and workers’ occupational health data were not obtained during the surveillance.

## Data availability statement

The raw data supporting the conclusions of this article will be made available by the authors, without undue reservation.

## Author contributions

CZ and JW conceived the study, analyzed the data, and drafted the manuscript. HW constructed the idea and reviewed and edited the manuscript. HZ planned the study, contributed to the discussion, and revised the manuscript. All authors contributed to the article and approved the submitted version.
